# Distribution of hepatitis C virus genotypes in patients infected by different sources and its correlation with clinical and virological parameters: a preliminary study

**DOI:** 10.1186/1476-5926-5-4

**Published:** 2006-10-02

**Authors:** Ali Kabir, Seyed-Moayed Alavian, Hussein Keyvani

**Affiliations:** 1 Tehran Hepatitis Center, Tehran, Iran; 2 Department of Gastroenterology, Baqyiatallah University of Medical Sciences, Iran; 3 Department of Virology, Iran University of Medical Sciences, Tehran, Iran; 4 Nikan Health Researchers Institute, Tehran, Iran

## Abstract

**Background:**

Information about genotypes and associated risk factors in hepatitis C virus (HCV) infected patients in Iran is limited. The aim of this study was to identify the HCV genotypes and associated risk factors in a group of HCV infected patients from Iran.

**Results:**

Genotyping analysis was performed in 156 patients with positive anti-HCV and HCV-RNA. Patients were questioned concerning documented risk factors. Genotypes 1 and 3 were found in 87 (55.8%) and 45 (28.8%) patients, respectively. The most frequent HCV subtype was 1a (37.8), followed by 3a (28.9%) and 1b (16.7%). There was no statistically significant difference between the risk factors analyzed and the acquisition of HCV infection. We further found that 18 (40%) and 17 (37.8%) patients that were intravenous drug users (IVDU) had genotype 1a and 3a respectively.

**Conclusion:**

Genotypes 3a and 1a in Iran are less prevalent in IVDU than in Europe and USA, but there is a high similarity between the pattern of genotype in IVDU in both Europe and United States, and Iran. However, in this case it can not be due to people migration among countries since history of travel abroad existed only in 6 cases (13.3%).

## Background

Chronic hepatitis C infection is now recognized as an important health problem [1]. Approximately 2–3% of the world population is infected with hepatitis C virus (HCV). HCV is one of the leading causes of liver failure and cancer, and the single most common indication for liver transplantation [2,3]. In Iran, the prevalence of HCV infection is about 0.12% in blood donors [4], but it is increasing. It seems that the prevalence of HCV infection is less than 1 percent in our general population, but the infection is emerging mostly because of problems such as intravenous drug use and needle sharing among drug addicts. HCV infection is the most prevalent cause of chronic hepatitis and cirrhosis in hemophiliac [5] and thalassemic patients [6], and patients with renal failure [7] in Iran. Different HCV isolates worldwide show substantial nucleotide sequence variability throughout the viral genome [8-11].

In the present study, we used PCR analysis with type-specific primers for identification of the HVC genomic typing, which enable the separation into six major genotypes (1 to 6) and a series of subtypes (e.g., a, b, c) [12-15]. These viral types and subtypes differ in their geographical distribution and antigenicity [8]. Types 1, 2 and 3 are distributed almost worldwide [16-22]. Types 4, 5 and 6 have been found in distinct geographical areas [12,20,21,23]. Interestingly, not only do the HCV genotypes seem to differ in nucleotide sequence and geographical distribution, but there is also evidence of biological differences between the three HCV genotypes. Patients with HCV subtype 1b have a poorer response to interferon alpha treatment [24-27]. Mode of transmission may also affect distribution of HCV genotypes [28-31].

Whereas the distribution of HCV genotypes in many countries is well documented, reliable data are still missing with respect to the frequency of the different HCV genotypes in Iran. We therefore conducted a study on patients with HCV infection, and correlated the mode of transmission, and the age, sex, and liver histology with the predominance of different genotypes. Accurate knowledge of HCV genotypes in our community is essential for successful future research into vaccine development and control strategy. Such information is needed to correctly formulate healthcare policies, prioritize interventions and allocate resources, accordingly. The aim of our study was to understand the main routes of transmission of HCV in our population, chosen from a referral clinic in Tehran, the capital of Iran.

## Results

The distribution of HCV genotypes evaluated in 156 patients by genotype screening [32] showed a major prevalence of HCV genotype 1 in 87 (55.8%) cases. Forty-five (28.8%) patients were infected with genotype 3, 2 patients (1.3%) with genotype 4, and 1 patient (0.6%) had mixed infection with genotypes 1 and 3. Genotyping was impossible in 21 patients. The distribution of subtypes of HCV genotypes related to age, sex, source of infection, Knodell's histological activity index (HAI), status of the liver disease, complete blood cell count (CBC), liver function tests (LFT), fasting blood sugar (FBS), triglyceride (TG), cholesterol, and serum protein electrophoresis given are compared in Table 1.

**Table 1:** Presentation of the 156 Iranian patients in relation to HCV-genotype.

**Presentation of patients**	**HCV-genotype**					**Sig**.
	**1a (N = 59)**	**1b (N = 26)**	**1a & 1b (N = 2)**	**3a^**c **^(N = 45)**	**4 (N = 2)**	
**Age (years)**^**a**^	37.5 ± 1.7	38.7 ± 2.4	46.5 ± 2.5	39.6 ± 1.8	46.5 ± 3.5	NS^d^
**Male/Female (%male)**	47/12 (79.7)	19/7 (73.1)	2/0 (100)	39/7 (84.8)	1/1 (50)	NS
**Transmission of HCV**						
***Post-transfusional***	24 (42.1)	10 (17.5)	1 (1.8)	15 (26.3)	0	NS
***IVDA***	18 (40)	5 (11.1)	0	18 (40)	0	NS
***Sexual***	16 (36.4)	6 (13.6)	0	19 (43.2)	0	NS
***Hemodialysis***	7 (36.8)	2 (10.5)	1 (5.3)	4 (21.1)	2 (10.5)	0.001
***Hemophilia***	3 (60)	0	0	2 (40)	0	NS
***Thalassemia***	5 (55.6)	2 (22.2)	0	1 (11.1)	0	NS
***Inmate***	12 (34.3)	2 (5.7)	0	17 (48.6)	0	0.041
***Travel abroad***	9 (40.9)	3 (13.6)	1 (4.5)	7 (31.8)	0	NS
***Hejamat*^**e**^**	8 (25)	6 (18.8)	1 (3.1)	14 (43.8)	1 (3.1)	NS
***Other risk factors***	10 (34.5)	5 (17.2)	0	7 (24.1)	0	NS
**Cirrhosis**	7	5	0	7	0	NS
**HAI**^**a, b**^	7.8 ± 1.1	10 ± 1.3	3 ± .0	8.6 ± 1.1	12 ± .0	NS
**AST (U/L)**	59.9 ± 5.8	67.8 ± 9.5	48.5 ± 12.5	71.1 ± 7.5	91.5 ± 75.5	NS
**ALT (U/L)**	73.6 ± 7.2	94.1 ± 14.8	40 ± 5	94.7 ± 10	139.5 ± 113.5	NS
**WBC (/ml)**	6866 ± 501	7405 ± 578	7000 ± 500	6993 ± 366	6500 ± 500	NS
**PLT (/ml)**	227281 ± 20455	194560 ± 10377	314000 ± .0	217391 ± 12168	244500 ± 22500	NS
**Hgb (g/dl)**	14.1 ± .33	14.5 ± .42	14.2 ± 2.6	14.5 ± .32	14.4 ± 2.2	NS
**FBS (mg/dl)**	105.4 ± 6.8	102.7 ± 9.4	97 ± 11	102.7 ± 8	106.5 ± 1.5	NS
**TG (mg/dl)**	142.3 ± 11.2	123.2 ± 10.7	176 ± 110	101.1 ± 6.9	215 ± 143	.016
**Cholesterol (mg/dl)**	159.7 ± 6.4	173.8 ± 9.1	185 ± .0	140.8 ± 7.3	186 ± 73	NS
**Serum albumin (g/dl)**	3.9 ± .08	4.1 ± .13	3.6 ± .25	4.1 ± .1	-	NS
**Serum protein (g/dl)**	7.4 ± .11	7.6 ± .18	6.4 ± .65	7.8 ± .13	-	.034
**Serum iron**	132.6 ± 13.8	118.4 ± 18.8	-	81.1 ± 8.8	110 ± .0	.024
**Weight (Kg)**	71.6 ± 1.9	70.5 ± 3.4	75.5 ± 14.5	72.7 ± 1.9	84.5 ± 2.5	NS
**Height (cm)**	170.8 ± 1.3	167.3 ± 2.1	169.5 ± 1.5	171.8 ± 1.2	163 ± 11	NS

^a ^Mean ± SE; ^b ^Histological activity index; ^c ^One patient had a mixed infection (3a(β)/1a); ^d ^Not significant; ^e ^A procedure in Iranian traditional medicine done by making shallow cuts on the trunk (upper back) and producing a suction effect that results in drawing blood from cuts (less than 100 cc). It is usually done by a non-physician, using non-standard instruments (done for healing or cure purposes). It is also named "cupping".

The alanino aminotransferase (ALT) level was not statistically different in cases with different genotypes, although it was slight higher in cases with genotype 4 and lower in cases with mixed genotype

There was any significant association between subtypes of HCV genotypes and the presence of anti-HBsAb (hepatitis B surface antibody), anti-HBcAb (hepatitis B core antibody), splenomegaly, ascitis, edema, cirrhosis, grade and stage of liver biopsy, and child score and status (inactive, chronic, cirrhotic and active) of the disease; revealing inexistence of any association between disease severity (grade, stage, child score and status of the disease) and different genotypes.

Only one patient with mixed infection with genotype 1a and 1b and two cases with genotype 3a had co-infection with hepatitis B virus (P < 0.001). Only one patient with mixed infection with genotype 1a and 1b and one case with genotype 1b had jaundice (P < 0.001). History of jaundice was seen more in cases with mixed infection with genotype 1a and 1b (2, 100%), 1a (12, 20.3%), 3a (6, 13%), and 1b (3, 11.5%). Any cases with genotype 4 had no history of jaundice.

From the 156 patients, only 135 cases had typeable genotypes. There were 8 cases with negative HCV RNA among 21 patients with a non-typeable genotype. We were unable to determine the genotype of the rest of 18 cases with the genotype-specific primer (GSP) method. One-hundred thirty patients had chronic hepatitis, either requiring treatment (89 patients) or not (41 patients). Other 26 patients were cirrhotic and needed supportive treatment. Duration of hepatitis for patients with both post transfusion and IVDU contamination were 10.6 ± 2.75 and 8.9 ± 3.53 years, respectively.

There was not any statistical significant association between the places of infection of the patients and genotype. However, genotype 4 was found only in north and west of country and mixed infection with genotype 1a and 1b only in center. In this study, the dominant genotype(s) in different regions of Iran consist: 1a, 1b and 3a in center and west, 1a and 3a in north and 1a in south and east (table 2). The geographic distribution of the patients with a typeable genotyping is summarized in table 2.

**Table 2:** The geographic distribution of the patients and their most prevalent genotypes.

**Infection place**	N° (Percent)*	**Prevalent genotypes: N° (Percent)**^**§**^				
		**1a**	**1b**	**3a**	**4**	**mix**
**Center**	77 (57.6)	33 (42.8)	15 (19.5)	27 (35.1)	0	2 (2.6)
**North**	18 (13.4)	7 (38.9)	2 (11.1)	8 (44.4)	1 (5.6)	0
**South**	1 (.7)	1 (100)	0	0	0	0
**East**	5 (3.7)	4 (80)	0	1 (20)	0	0
**West**	31 (23.1)	13 (41.9)	8 (25.8)	9 (29)	1 (3.2)	0
**out of Iran**	2 (1.5)	1 (50)	1 (50)	0	0	0

*Percents of cases from different geographic parts.

^§^Percents of the different genotypes in different geographic parts.

## Discussion

Genotyping is important because it provides information as to strain variation and potential association with disease severity. In addition, it is of epidemiologic value because it sheds light on whether prevalent HCV strains are similar to that endemic in a certain region, such as herein in the Middle East.

In comparison with studies made in Iran's neighbor countries, it can be understood that the most common genotype of Yemen, Kuwait, Iraq, and Saudi Arabia is type 4 [12]. However, subtype 1b in Turkey [33] or western border of Iran and subtype 3a in Pakistan or eastern border of Iran are more prevalent [34]. Although genotype 4 is found almost exclusively in Middle East and western countries [35], this genotype is uncommon in our country and related to different route of contamination such as dialysis, minor surgery, piercing or hejamat (see footnote in Table 1), and not to transfusion, intravenous drug abuse (IVDA) or sexual contacts. Another study showed that genotype 4 is over-represented among hemodialysis patients in Tehran [36]. However, we can not rule out any definite conclusion on genotype 4 transmissions with only 2 patients.

On the other hand, subtype 1b is more prevalent in Turkey and Russia [37] (west and north of Iran). This subtype is one of the common genotypes in Iran as the present study and some other limited studies have previously shown [38,39]. This subtype is more frequently seen in cases with history of hospitalization (17 cases, 60.7%), major surgery (15 cases, 53.6%), dental surgery (12 cases, 42.9%), transfusion (11 cases, 39.3%), alcohol consumption and minor surgery (each one in 8 cases, 28.6%), whereas making 16.7% as a total.

Although the previous studies have had lower sample sizes, their results are similar to our study. They had concluded that subtypes 1a, 3a, and 1b are the most common types respectively and that type 4 is rare [38,39].

A similarity was observed between our country and both Pakistan (the eastern neighbor of Iran) and India, in which the genotype 3 is very prevalent and genotype 2 is very rare, like in our country [40,41]. Other studies in Iran have shown the absence of genotype 2 as well [36,38,42]. We think that this can be due to the high rate of immigration from these countries to Iran, especially when considering the fact that the prevalence of HCV infection in these countries is higher than Iran. However, more investigations are needed for establishing a definitive judgment.

Genotypes 3a and 1a are more prevalent in IVDU in Europe and USA [28-31,43]. In the present study, 18 (40%) and 17 (37.8%) patients with IVDA had genotype 1a and 3a respectively. It seems that there is a high similarity between the pattern of genotype in IVDU in Europe and United States when compared with Iran. However, it can not be due to migration of these people to these countries because the history of travel abroad was only seen in 6 cases (13.3%).

In the present study, and concerning the route of HCV transmission, most of the patients seem to have multiple routes of contamination which limits the conclusion on relationship between genotype and route of contamination. The inmate route of contamination may be due to IVDA, as it is observed in other countries. However, genotype 3 was more frequent in IVDU. Genotype 4 was also seen only in patients undergoing hemodialysis and/or hejamat.

There was no difference in genotypes in terms of age and sex of the patients. This pattern is different when compared to reports from developed countries, where lifestyles among young adults seem to have influenced the molecular epidemiology of HCV by the introduction of subtype 1a and 3a from USA and Southeast Asia into their young drug addicts [44].

Our results are in accordance with the predominance of genotype 1 observed in most countries worldwide [12,16,20,45,46]. With respect to the zero frequency of genotype 2, our data differ from those published for patients in the United States, Europe, and even Asia, which showed a different prevalence of genotype 2 [19,24,31,35,47].

## Conclusion

Genotypes 3a and 1a in Iran are less prevalent in IVDU when compared with Europe and USA. Moreover, it seems that there is a high similarity between the pattern of genotype in IVDU in Iran when compared with those in Europe and United States. However, we think that this occurrence can not be due to migration phenomena among involved countries because of history of travel abroad existed only in 6 cases (13.3%).

## Materials and methods

We evaluated all the 156 cases with hepatitis C infection (125 male, 31 female; mean age 38.9 ± 1, age range 14–71 years) referred to the Tehran Hepatitis Center from June 2002 to May 2003, consecutively. The diagnosis of chronic hepatitis C was made on the basis of the presence of anti-HCV antibodies in both sera detected by third-generation commercially available enzyme-linked immunosurbent assay (ELISA) kits (ETI HCV K-3, DiaSorin, Spain) and HCV RNA detected qualitatively by reverse transcriptase polymerase chain reaction (Amplicore II, Roche, NJ, USA).

CBC, LFT and serum protein electrophoresis were performed, and FBS, TG, and also cholesterol were checked in all patients. These were questioned concerning documented risk factors acting as main infection routes, namely IVDA, blood transfusions, acupuncture or tattoos, extra marital sexual contact, hemodialysis, hemophilia, thalassemia. Other risk factors were also checked.

At the time of the study 89 patients had chronic hepatitis requiring antiviral therapy. Twenty-six cases were cirrhotic and 41 patients did not need treatment. Liver biopsy was performed in 72 patients. Chronic hepatitis was diagnosed in 57 and liver cirrhosis in 12 patients. No specific pathologic change occurred in only 3 patients. The histological finding was further graded according to the HAI of Knodell et al. [34]. The mean HAI score was 8.7 ± 0.6 (range 1–20).

The mean ± standard error (SE) was used for the description of quantitative variables. Whereas the Student *t*-test and one-way ANOVA were used for comparing quantitative variables, the chi-square test was used for comparisons involving categorical variables. Differences or correlations with P < 0.05 were considered statistically significant. SPSS software (Version 11.5, SPSS Inc. Chicago, Illinois, USA) was used for the analysis. The study protocol conforms to the ethical guidelines of the 1975 Declaration of Helsinki.

For the genotype specific primer approach, viral RNA was extracted from 100 μl of HCV positive patients' serum, using guanidine throcyanate and isopropanol. Precipitated RNA was washed with 70% ethanol and then dissolved in 200 μl TE buffer. Five μl of the dissolved RNA was immediately reverse transcribed by using random hexamer. Genotyping was performed as described previously [32].

## Competing interests

The author(s) declare that they have no competing interests.

## Authors' contributions

HK carried out the molecular genetic studies, the sequence alignment and the immunoassays, and also drafted the manuscript. AK and S-MA conceived and coordinated the study, helped to draft the manuscript, and made the statistical analysis. All authors read and approved the final manuscript.
